# Cost-effectiveness of preventing dental caries and full mouth dental reconstructions among Alaska Native children in the Yukon–Kuskokwim delta region of Alaska

**DOI:** 10.1111/jphd.12141

**Published:** 2016-03-15

**Authors:** Charisma Y. Atkins, Timothy K. Thomas, Dane Lenaker, Gretchen M. Day, Thomas W. Hennessy, Martin I. Meltzer

**Affiliations:** 1 Health Economics and Modeling Unit, Division of Preparedness and Emerging Infections, National Center of Emerging & Zoonotic Diseases, Centers for Disease Control & Prevention (CDC), Atlanta, GA, USA; 2 Alaska Native Tribal Health Consortium, Anchorage, AK, USA; 3 Yukon-Kuskokwin Health Consortium, Bethel, AK, USA; 4 Arctic Investigations Program, Division of Preparedness and Emerging Infections, National Center of Emerging & Zoonotic Diseases, Centers for Disease Control & Prevention (CDC), Atlanta, GA, USA

**Keywords:** dental caries, FMDR, dental interventions, cost-effectiveness, cost saving

## Abstract

**Objective:**

We conducted a cost-effectiveness analysis of five specific dental interventions to help guide resource allocation.

**Methods:**

We developed a spreadsheet-based tool, from the healthcare payer perspective, to evaluate the cost effectiveness of specific dental interventions that are currently used among Alaska Native children (6-60 months). Interventions included: water fluoridation, dental sealants, fluoride varnish, tooth brushing with fluoride toothpaste, and conducting initial dental exams on children <18 months of age. We calculated the cost-effectiveness ratio of implementing the proposed interventions to reduce the number of carious teeth and full mouth dental reconstructions (FMDRs) over 10 years.

**Results:**

A total of 322 children received caries treatments completed by a dental provider in the dental chair, while 161 children received FMDRs completed by a dental surgeon in an operating room. The average cost of treating dental caries in the dental chair was $1,467 (~258,000 per year); while the cost of treating FMDRs was $9,349 (~1.5 million per year). All interventions were shown to prevent caries and FMDRs; however tooth brushing prevented the greatest number of caries at minimum and maximum effectiveness with 1,433 and 1,910, respectively. Tooth brushing also prevented the greatest number of FMDRs (159 and 211) at minimum and maximum effectiveness.

**Conclusions:**

All of the dental interventions evaluated were shown to produce cost savings. However, the level of that cost saving is dependent on the intervention chosen.

## Introduction

Tooth decay or dental caries is one of the most common chronic conditions among American children as reported by the American Academy of Pediatrics Children’s Oral Health Initiative (1). In April 2008, the Arctic Investigations Program of the Centers for Disease Control & Prevention (CDC-AIP) was informed of high rates of dental caries among Alaska Native (AN) children residing in the Yukon–Kuskokwim Delta (YKD) region of Alaska (2). AN children aged 48-60 months had a mean of 7.32 decayed, missing, and/or filled primary teeth (dmft) (3). Additionally, approximately 400 full mouth dental reconstructions (FMDRs) were performed on AN children less than six years of age (i.e., 72 months) in 2007; approximately 12.2 percent or 1 in 8 of the total population of less than 6 years (*n*=3,000) (2-4). FMDRs, which often are done under general anesthesia, typically involve multiple extractions of carious teeth and restorative procedures such as fillings or crown placement. These procedures frequently require the hospitalization of young children with extensive treatment needs, and the costs include use of dental providers, dental surgeons, operating rooms, medications, and travel and accommodations for the child and their parents/guardians. These dental treatments incur considerable cost to Medicaid and other healthcare payers. The use of interventions that can notably reduce the rate of dental caries in children would not only reduce the number of children requiring treatment, but would also alleviate the cost burden on the healthcare system. CDC was asked by the YK Dental program for technical assistance in determining whether current interventions were cost beneficial and effective in reducing the number of carious teeth in YKD children.

We examined, from the healthcare payer (i.e., Medicaid) perspective, the economic impact of 5 interventions currently used among YK children to reduce the economic burden of treating dental caries among children (6-60 months) in the YKD region. These data may aid public health officials and primary dental care providers to choose those interventions likely to have the greatest impact in reducing rates of dental caries in this population.

## Methods

We used Microsoft Excel© 2010 to develop a simple Excel spreadsheet based tool (Appendix I) to evaluate the economic impact associated with implementing 5 different, currently used or potential dental interventions in the YKD for AN children per age cohort (6-12 months, 13-24 months, 25-36 months, 37-48 months, and 49-60 months). In consultation with dental providers in the YKD, specific interventions were chosen to be included in our analysis because they were either already being used in the population or they were expected to be the most successful in preventing the development of future caries in the population. These interventions were water fluoridation, dental sealants, fluoride varnish applications, home tooth brushing with fluoride toothpaste, and conducting initial dental exams on children less than 18 months of age with parents receiving parental counseling. We also developed a methodology to evaluate the number of adverse health outcomes (i.e., dental caries and FMDRs) prevented and the cost effectiveness of preventing those outcomes (i.e., $/health outcome averted prevented) due to the implementation of each dental intervention. Our study population comprised of those patients evaluated or treated for dental caries by a dentist or other dental provider in a tribally-run hospital or clinic in the YKD. Our model is unique in that it represents only Alaska Native Children in the YKD region; and thus the results cannot be generalized to represent all children in other states and territories, without implementing considerable changes to the model inputs. All analyses were assessed using minimum and maximum effectiveness at current and ideal population coverage. We used, with the exception of water fluoridation, the 2013 Alaska Medicaid Dental Fee Schedule (5) to calculate the cost of each intervention. For water fluoridation, we used the 2012 Rural Alaska Water Fluoridation Cost Calculations to generate total cost of implementing and maintaining a typical water fluoridation system in the YKD region. Our study perspective was that of the health care payer (i.e., Alaska’s Medicaid program), and we discounted all outcomes and costs, where appropriate, at 3 percent per year over a 10 year time-frame, using US 2011 dollars.

### Population

The YK region is composed of 48 communities with a total population of approximately 25,000, of which 85 percent are Yup’ik Eskimo people (3). The largest community in this region is Bethel, with a total population of nearly 6,300 people. Approximately 11 percent (~2,575) of the YK population is comprised of children ages 6-60 months (5). In 2011, 1,536 children (6-60 months) were seen for dental services (Dental Procedural Visits, Number of Children Seen for Caries Treatment by Intervention. 2011. YKHC) [Appendix I].

We began our evaluation by calculating the current and ideal population coverage for each intervention. Current population coverage is the percentage of the population presently receiving a specific intervention; whereas ideal population coverage is the maximum percentage of the population capable of receiving the intervention.

We determined the current population coverage for each intervention using the following formula:

(2.1) $$=\frac{\mathrm{Current population coverage}\;(\mathrm{percent})\;\mathrm{of children receiving each Intervention}}{\mathrm{Total child population}}$$

As reported by the US Census, the total child population (6-60 months) within the YKD region is 2,575. The number of children receiving each intervention varied. Data were obtained from the 2011 Yukon-Kuskokwim Health Corporation (YKHC) Dental Services database (Dental Procedural Visits, Number of Children Seen for Caries Treatment by Intervention, 2011, YKHC). For simplicity, we assumed that all interventions had an ideal population coverage of 100 percent of recommended age groups, with the exception of water fluoridation. The current population coverage for water fluoridation was calculated in the same manner as that of the other interventions. Five communities (929 children) currently receive fluoridated water generating population coverage of 36 percent. Unfortunately, not all communities in the YK region have the capacity to receive fluoridated water; therefore the ideal population coverage was based on the maximum number of communities that could be fluoridated. The Water Fluoridation Status of 2012 (6), reported that only 17 additional communities (830 children) in the YKD have the capability of establishing and receiving piped water. Thereby, the maximum number of children capable of receiving water fluoridation is 1,759 (22 communities in total) leading to an ideal population coverage of only 68 percent.

### Treatment of dental caries

Dental caries, or cavities as they are more widely known, are caused by bacterial infections that destroy the tooth enamel resulting in tooth decay (YKHC Quality Systems Incorporated (QSI) Electronic dental record database (“Clinical Product Suite”, 2011). We assumed that children in the YKD were treated for dental caries either by the local dentist or dental provider during a dental visit or by a dental surgeon in a hospital operating room.

#### Caries treatment in a local dentist office

In 2011, 1,536 YK children were evaluated or treated for caries in a dental chair (i.e., all procedures done either in a dental office or during a dental visit by the local dentist or dental health provider). Of those being seen for dental treatment in a dental chair during a dental visit, 156 children received at least one crown, 166 received at least one filling, and 188 children received a combination of both crowns and fillings. We calculated the average number of crowns per child as 4.54 and the number of fillings per child as 3.18 producing a total number of 708 crowns and 528 fillings, respectively (Table 1). The total average mean number of crowns and fillings is 7.73 per child with a total of 1,453 teeth expected to be treated for crowns and fillings. The annual incidence of children (6-60 months) receiving a crown and/or filling by a local dental practitioner in the YKD region was 7.3 percent.

The cost of caries treatment typically includes an oral exam ($66.98), X-ray ($89.08), the mean cost for placement of a stainless steel crown on a primary tooth ($199), mean cost for resin-based anterior filling ($214), and the mean cost of a child receiving both crown and fillings during one visit ($1,050). Oral exam and X-ray costs are the customary Medicaid reimbursement fees associated with any type of dental treatment. The mean cost for stainless steel crowns, resin-based fillings, and cost of both crown and filling was weighted by the number of children receiving a specific type of treatment (i.e., crowns only, fillings only, or combination of fillings and crowns) compared to the total number of children being seen for the placement of fillings and/or crowns (Appendix II). In 2011, 90 percent of all fillings completed were resin-based with 63 percent treated using 1-surface resin-based anterior fillings, 24 percent used 2-surface, 9 percent used 3-surface, and 4 percent used four or more surfaces (Arctic Investigation Program. YK Dental Frequency, Number of Dental Procedures Completed in YK. 2011 and YKHC Quality Systems Incorporated (QSI) Electronic dental record database (“Clinical Product Suite”, 2011). Because our analysis is based on what is currently being exhibited in the YKD, we used resin-based fillings only to estimate the treatment cost. A child receiving both crowns and fillings was assumed to receive equal number of both because we did not have data detailing the exact number of crowns and fillings done in children that received both treatments during one visit. Thus, the total mean treatment cost for treating children with carious teeth in the dental chair requiring at least 1 crown, filling, or combination of both is the sum of all the exam costs and all the mean treatment costs (~$1,400), calculated using Equation 2.2.

(2.2) $$\begin{array}{r} \mathrm{Total Weighted Caries Treatment Cost}\;(\$)\;\mathrm{per child}\\ =\mathrm{weighted mean crown}\;\$\;(199)\\ +\;\mathrm{weighted mean filling}\$(214)\\ +\;\mathrm{weighted mean crown}\&\mathrm{filling}\$(1050) \end{array}$$

The total annual estimated cost of treating children with dental caries requiring a filling, crown, or a combination of both fillings and crowns (188 children) is $275,890 (Table 1).

#### Caries treatment in hospital operating room

To perform treatment safely, effectively, and efficiently, the practitioner caring for a child with extensive dental disease often requires FMDRs under sedation or general anesthesia. FMDRs frequently involve multiple extractions, restorations (i.e., crowns, fillings), and pulpotomies, thus making them quite costly. The success of these restorations may be influenced by the child’s level of cooperation during treatment, and general anesthesia may provide better conditions to perform these procedures (7). Instances in which the placement of crowns and/or fillings on the carious teeth have been completed while the child is under general anesthesia has allowed the dentist to perform all the necessary services during one visit; thus preventing the child from having to return to complete the caries treatment. In 2011, 161 FMDRs were performed on children, 6-60 months of age, living in the YKD (6.3 percent of children 6-60 months).

The cost of treating children with FMDR includes travel (~$1,500), use of personnel involved in performing the procedure (~$1,500), and operating room rental plus prescription drugs, and anesthesia (~3,198). The median number of teeth extracted and pulpotomies completed per FMDR was 4 and 5, respectively. We used the following Equation 2.3 to calculate the total cost of completing FMDRs ($9,349/child). Refer to Appendix II for a complete set of equations used to calculate treatment costs.

(2.3) $$\begin{array}{c} \mathrm{Total Mean FMDR Cost}\;(\$)\;\mathrm{per child}=\mathrm{Travel}\$+\;\mathrm{Personnel}\$\\ +\;\mathrm{Operating Room}/\mathrm{Pharmaceutical}\$+\;(\mathrm{Median}\#\mathrm{of Crowns per child}\times \;\mathrm{Crown}\$)\\ +\;(\mathrm{Median}\#\mathrm{of fillings per child}\times \;\mathrm{Filling}\;\$\mathrm{per tooth})\\ +\;(\mathrm{Median}\#\mathrm{of Teeth Extracted}\times \;\mathrm{Tooth Extraction}\$)\\ +\;(\mathrm{Median}\#\mathrm{of Vital Pulpotomy}\times \;\mathrm{Pulpotomy}\$)\\ +\;(\mathrm{Median}\#\mathrm{of Bitewings}/\mathrm{Films}\times \;\mathrm{Bitewings}/\mathrm{Films}\$)\\ +\;\mathrm{Associated Additional}\$ \end{array}$$

The overall estimated cost of completing all FMDRs on the 161 YK Delta children (6-60 months) in 2011 was $1.5 million (Table 1).

### Interventions: effectiveness and costs

In response to the request by the YK Dental program for technical assistance in determining whether specific interventions were cost beneficial and effective in reducing the number of carious teeth in YKD children, we examined the cost effectiveness of 5 currently used or potential preventive interventions among children (6-60 months) in the YK region. These interventions were water fluoridation, dental sealants, fluoride varnish, home tooth brushing with fluoride tooth-paste, and initial dental exam with parental counseling before 18 months of age. We used a range of effectiveness values (minimum and maximum), along with current and ideal population coverage and program costs for each intervention (Table 2). Current population coverage is defined as the “present day” percentage of persons receiving each intervention; while the ideal population coverage is the maximum percentage of the population who could receive the intervention. With the exception of water fluoridation, all interventions had an ideal population coverage of 100 percent of the recommended age groups.

- Water Fluoridation
  Adjusting the level of fluoride in the community water systems results in a 26-35 percent reduction in tooth decay among children receiving lifelong exposure to fluoridated water (6,8). Other estimates based earlier YK dental reviews suggests 18-40 percent reduction in tooth decay among children receiving community fluoridated water. Due to a number of reasons, such as lack of suitable infrastructure and problems caused by permafrost, not all communities in the YKD are capable of receiving a piped water fluoridation system. Currently, only five (5) communities (9) in the YK region, with a total population of 929 children (4), have a fluoridated water system, resulting in current population coverage of 36 percent. We determined that out of the 48 YK communities, an additional 17 communities with 830 children aged 6-60 months, have the capacity to receive fluoridation in the near future. (4). Extending water fluoridation to those communities would increase coverage to 68 percent of all children (6-60 months) in the region.
  The cost to fluoridate a community includes a one-time only start-up cost (10) of $7,090 ($5,500 for installation and $1,590 for travel), as well as annual operator’s fees above their own salary of $2,080 and an annual renewal cost of $1,545. Thus, the overall annual cost per community using fluoridated water is $3,625 (Table 2).
- Dental Sealants
  In populations at high risk of dental caries, the American Dental Association recommends children should receive sealants on both their primary and permanent molars (11,12). Previous effectiveness studies suggest that 74 percent of primary molars that receive sealants remain caries free (13). Recent studies suggest that the placement of sealants on the permanent molars of children results in a reduction of caries incidence ranging from 71 to 78 percent (14,15). Children’s first of 8 primary molars typically erupt between 13 and 19 months of age (16). Local Alaskan dentists recommend that children should receive sealants on their primary molars before their third birthday. Ideally, 4 molars would be sealed between 12 and 24 months and additional 4 between 24 and 36 months. In an effort to determine the total number of caries reduced by sealants, we multiplied the percent effectiveness by the ratio of molars to the total number of teeth present in a child’s mouth (8/20). We chose to use resin-based sealants because resin-based sealants were completed most often on the children in the YKD, according to the YK dental frequency database for services rendered. While we understand, it is difficult to maintain a dry field when placing sealants in young children, which can reduce retention and ultimately sealant effectiveness; we believe using materials that represent current practice will yield more valid estimates of cost effectiveness. During 2011, a total of 250 children 6-60 months received at least one sealant (Arctic Investigation Program. YK Dental Frequency, Number of Dental Procedures Completed in YK. 2011) (20) yielding an overall current population coverage of 10 percent. We assumed, for ideal coverage, that a total of 8 molars would be sealed in 100 percent of all children when they are aged 12-36 months (each child has 4 molars sealed per year).
  The Medicaid reimbursement cost for applying dental sealants is $49.68 per tooth (5).
- Fluoride Varnish Applications
  A fluoride varnish application consists of having a 5 percent sodium fluoride varnish solution applied in small amounts directly on tooth surfaces and only requires 1-2 applications per child per year for efficacy (18). Studies suggest that topical fluoride products should be placed on the primary teeth of children during their well child visits from the age of 9-30 months (11,18). In 2011, Slade et al. conducted a trial amongst the aboriginal child population in Australia and found caries reduction between 18-24 percent (19). Earlier studies suggested there was approximately 20-40 percent reduction in caries incidence when varnish was used appropriately (20,21). Furthermore, a meta-analysis of three studies assessing the effects of fluoride varnish on children’s deciduous teeth (i.e., baby teeth, temporary teeth, primary teeth) suggests a 33 percent reduction in decayed, missing, or filled tooth surfaces (22). During 2011, the YKHC Dental Database reported that 1311 children aged 6-60 months received varnish applications (annual mean of 1.68 applications per child per year). The overall current population coverage is 51 percent and we assumed an ideal population coverage of 100 percent of children 6-60 months.
  The Medicaid reimbursement cost for varnish application during one dental visit is $28.50 (5).
- Home Brushing with Fluoride Toothpaste
  American Academy of Pediatric Dentistry (AAPD) and the American Dental Association (ADA) guidelines recommend that children should brush their teeth with fluoride toothpaste twice daily (23) to assist in the prevention and control of caries. Fluoride use is recognized by both organizations as a safe and highly effective strategy for preventing and controlling caries. Dentists recommend that children younger than 3 years should use a “smear” or “rice” size amount (~.1mg) of fluoride, while children aged 36-60 months should use a small amount (~.25mg) of fluoridated toothpaste to brush their teeth twice daily and be assisted by an adult to help them in their home brushing. Wright et al. suggest that the daily practice of tooth brushing using the appropriate amount of fluoride toothpaste is effective in the reduction and control of dental caries (23,24). A 2008 study of 5 towns in the YKD, found that 91 percent of all children (6-60 months) brushed their teeth daily and of those, 55 percent of children aged 48-60 months brushed their teeth at least twice a day [Dental Epi-Aid, Toothbrush Practices in 5 YK Delta Towns. 2008. Unpublished CDC data.]. Ellwood *et al*. illustrated that the consistent delivery of fluoride toothpaste and toothbrushes to children at 3-month intervals was effective in reducing caries incidence by 16 percent (25). More recent studies have revealed that daily use of fluoride toothpaste on the primary teeth of children could prevent the occurrence of dental caries by between 21 and 28 percent, with a prevention factor of 24 percent (26,27). We assumed that, in an ideal situation, all children aged 6-60 months of age would be given toothbrushes. We also assumed that toothbrushes would be replaced every 3 months (i.e., 4 toothbrushes/child/ year), and at least two tubes of medium sized toothpaste, approximately 11 ounces, would be used (i.e., 8 tubes per year). YK children receive their supply of toothpaste and toothbrushes either during their well child visits in the dental office or during home visits from dental assistants.
  We assumed, for each child, a cost of $5 per toothbrush and $3 per tube of toothpaste (i.e., $44/child/year).
- Initial Exam with Parental Counseling
  YK dentists recommend that children receive an initial exam by a dental health provider with parental counseling prior to 18 months of age. There is limited information as to the effectiveness of conducting dental examination and providing parental counseling to prevent dental caries in preschool children (11). However, Feldens *et al*. reported that parental counseling can reduce caries by 22 percent (28). Other studies suggest that severe early childhood caries incidence can be reduced by as much as 32 percent (29,30). In 2011, there were 570 children ages 6-18 months residing in the YKD (4). Since 162 children (6-18 months) received initial dental exams prior to 18 months, current population coverage is 8 percent. We assumed an ideal population coverage of 100 percent among children 6-18 months.
  Medicaid reimburses $66.98 for an initial examination (5). We assumed that each child may only receive one initial examination, with parental counseling.

### Dental intervention program cost

Intervention program costs (Table 3) were calculated using the Medicaid reimbursement fee associated with supplying each intervention to the suggested population (current and ideal population coverage), number of children receiving the intervention, mean number of teeth or applications used, and recommended usage to reach full effectiveness for each age cohort. The total costs across all age groups were then summed.

We calculated the undiscounted and discounted costs for each dental intervention, with the exception of water fluoridation (Refer to earlier subsection), at Year 1 and Year 10 using Equations 2.4 and 2.5 below.

(2.4) $$\begin{array}{r} \mathrm{Current Cost}\;(\$)\;=\mathrm{Reimbursement}\$\times \;\mathrm{annual of children receiving each Intervention}\\ \times \;\mathrm{Average No. of Teeth}/\mathrm{Applications Treated} \end{array}$$

(2.5) $$\begin{array}{r} \mathrm{Ideal Cost}\;(\$)\;=\mathrm{Reimbursement}\$\times \;(\mathrm{Ideal Population percent}\\ \times \;\mathrm{annual of children receiving each Intervention}\\ \times \;\mathrm{Recommended Eff Usage}) \end{array}$$

The average numbers of teeth treated or applications done were obtained from the 2011 YKHC QSI Electronic Dental Record (YKHC Quality Systems Incorporated (QSI) Electronic dental record database (“Clinical Product Suite”, 2011). Refer to spreadsheet tool in Appendix I (Arctic Investigation Program. YK Dental Frequency, Number of Dental Procedures Completed in YK, 2011) for the annual number of children served under each intervention for each age cohort (6-60 months). Each age group had a specific number of teeth treated or applications done. We calculated the average mean value across all age cohorts. The recommended number of dental applications needed for the intervention to be effective was obtained from literature reviews and local dental practitioners. For instance, local YK dentists suggest that children should receive up to 8 dental sealants on their primary molars prior to their third birthday, since all of their primary molars should have erupted by that point. Dentists also recommend that children should receive at least 2 varnish applications per year during well child visits (9, 12, 15, 18, 24, and 30 months).

### Adverse dental health outcomes prevented

One of our primary objectives was to determine whether specific dental interventions could be used to reduce the total number of adverse dental health outcomes, such as dental caries and FMDRs, observed. We calculated the number of carious teeth and FMDRs prevented using the average number of carious teeth per child and the population covered (per intervention type) as shown in Equation 2.6 and 2.7 and Figure 3:

(2.6) $$\begin{array}{l} \mathrm{No. of Caries Prevented per year}\\ =(\mathrm{Current or Ideal Pop Covered}\\ \times \;\mathrm{Proportion of children receiving Crowns and}/\mathrm{or Fillings}\\ \times \;\mathrm{Avg No. of Carious Teeth per Child})\\ \times \;\mathrm{Effectiveness Rate}\;(\min\&\max) \end{array}$$

(2.7) $$\begin{array}{r} \mathrm{No. of FMDRs Prevented per year}\\ =(\mathrm{Current or Ideal Pop Covered}\\ \times \;\mathrm{percent of children expected to have a FMDR})\\ \times \;\mathrm{Effectiveness Rate}\;(\min\&\max) \end{array}$$

The population covered is the number of children receiving each intervention as reported in the Methods section, whereas the ideal population coverage for interventions, with the exception of water fluoridation, was the total number of children in the YKD in the correct age group to receive the intervention. In 2011, amongst children (6-60 months) in the YKD being seen for dental treatment, 12.2 percent received either a crown and/or filling. A total of 161 FMDRs were completed during 2011, indicating that 6 percent of the total population (6-60 months) received a full mouth reconstruction during the year. We assumed that each child could receive only one FMDR in a given year; thus the proportion receiving FMDR is also 6 percent for each intervention. For instance, the current number of children 12-36 months using dental sealants in 2011 was 250; therefore, the number of FMDRs completed on children using dental sealants was approximately 15.

### Cost effectiveness of preventing adverse dental health outcomes

Our final step was to determine which intervention would have the greatest impact on reducing the number of carious teeth and FMDRs using cost-effectiveness analysis. Next, we calculated the total adverse health outcomes prevented in the population, at minimum and maximum effectiveness, using Equations 2.6 and 2.7. Using these values, we then applied the cost associated with treating children for dental caries and FMDRs, separately. Using these costs we estimated which intervention would cost the least, but prevent the greatest number of adverse health outcomes (Figure 2) for both.

Lastly, we calculated the discounted cost effectiveness ratio (CER) for current and ideal population coverage using Equation 2.8.

(2.8) $$\mathrm{CER}=\frac{\mathrm{Program}\$-\mathrm{Prevented Adverse Health Outcome}\$}{\mathrm{Prevented Adverse Health Outcomes}}$$

where CER is expressed as the difference between program cost and cost per health outcome prevented divided by the number of health outcomes prevented due to the use of the intervention.

## Discounting

We applied a discount rate of 3 percent to all outcomes (e.g., cost, dental caries prevented, general anesthesia prevented). Discounting was used to estimate the future value and cost of the dental interventions. We applied a discount rate of 3 percent to all outcomes (e.g., cost, dental caries prevented, general anesthesia prevented). Discounting was used to estimate the present value (PV) and cost of the dental interventions using their current present day undiscounted values. The formula for discounting (Equation 2.9) is as follows:

(2.10) $$\mathrm{Discounting}\;(\mathrm{PV})=\frac{\mathrm{Undiscounted Annual Cost or Outcomes}\;(\mathrm{at time}\;0)}{{\left(1+\;\mathrm{rate}\right)}^{\wedge }\mathrm{timeframe}}$$

where:

Time 0 = Present day estimated calculated value for each intervention
Rate = 3 percent (universal health evaluation standard) Timeframe5 length of time intervention used

## Results

**Figure 1:**
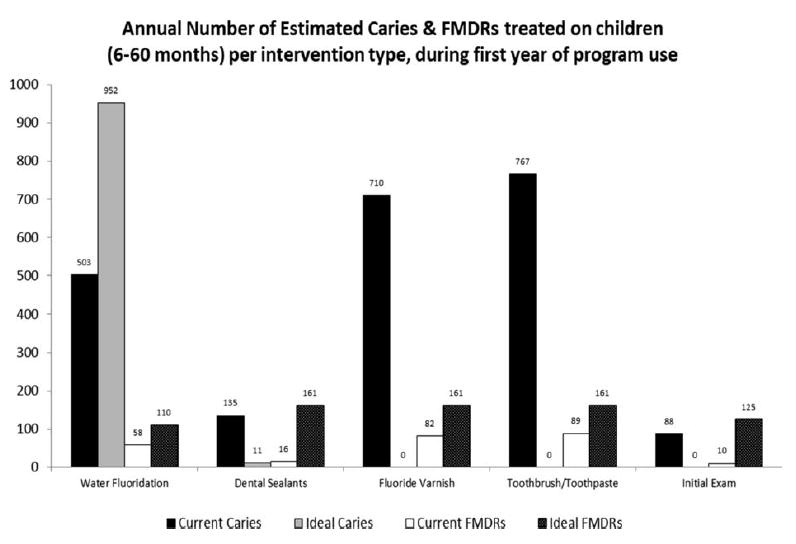
Annual number of caries treated and FMDRs completed by intervention type. The values presented display the annual number of expected caries and the number of FMDRS completed in children by intervention type. Expected caries is calculated using the product between caries incidence, population receiving treatment, and the average number of caries (crowns and/or fillings) per child. The average number of caries per child is 1.71. We assumed that each child could only receive one FMDR per year; thus the annual number of FMDRS is the product between the population of children receiving the intervention and the FMDR incidence per child. Annual caries incidence for children receiving a crown and/or filling was 7.3 percent, while the annual FMDR incidence was 6.3 percent.

**Figure 2:**
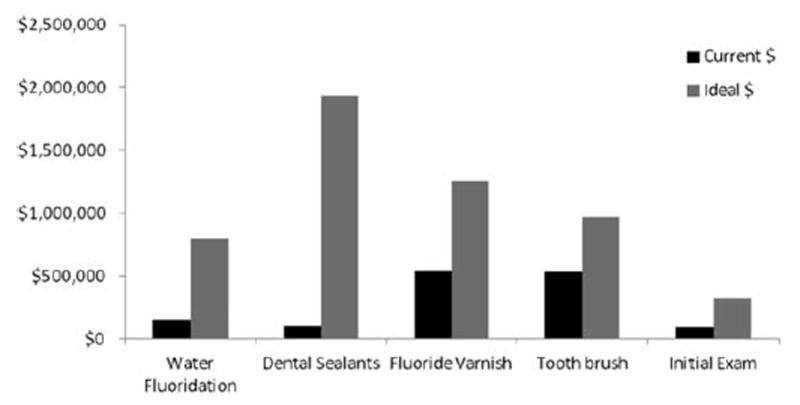
Total discounted program costs over a 10-year timeframe. Program costs are discounted using a rate of 3 percent. We calculated the total program costs of using a specific intervention for the full implementation timeframe of 10 years.

Treating children with caries in the dental office cost approximately $1,467 per child ($275,890 annually), whereas the cost of completing FMDRs was $9,349 per child ($1.5 million annually). We first estimated number dental caries and FMDRs expected to occur, both annually and over the full 10 year timeframe. Next, we applied a discount rate of 3 percent to calculate the total program cost and adverse health outcomes prevented for each intervention (Figures 1 and 2). We calculated the undiscounted and discounted cost for each intervention at the current and ideal population coverage using Equations 2.4 and 2.5 in Tables 3 and 4. For comparative analysis, we provided Year 1 undiscounted estimates for each intervention in Table 4.

- The current undiscounted first year cost of supplying water fluoridation to the 5 communities already receiving water fluoridation is $18,125 and the total undiscounted first year cost of implementing water fluoridation to all communities capable of receiving water fluoridation is $200,280. The discounted 10 year cost of fluoridating all 22 communities is $797,303.
- The annual undiscounted current year cost of applying dental sealants is $12,420 with the maximum undiscounted cost of increasing coverage to 100 percent totalling $226,938. The total 10-year discounted cost is approximately $1.9 million.
- The current coverage undiscounted cost of applying fluoride varnish to YK children is $62,923 and the maximum cost being $146,775 at 100 percent coverage. The discounted 10 year cost is approximately $1.3 million.
- The current undiscounted annual cost of providing fluoride toothpaste and toothbrushes is $62,135 and the maximum undiscounted annual cost is $113,000. The total discounted 10 year cost is $966,472.
- The current undiscounted cost of providing initial exams to children prior to 18 months of age is $10,851 with the maximum current year cost at 100 percent coverage being $38,179. The total discounted 10 year cost is $325,671.

We then calculated the number of caries and FMDRs prevented at minimum and maximum effectiveness for both current and ideal population coverage. For instance, during Year 1 (Figure 1), there were a total of 929 children currently receiving water fluoridation with 68 children expected to have caries. However, the application of the effectiveness rates prevented between 136 (minimum effectiveness) and 184 (maximum effectiveness) caries. Once the discount rate was applied, the number of caries prevented ranged from 132-178 at current coverage levels during the first year of water fluoridation implementation. Likewise, the number of children that could be ideally covered under water fluoridation during Year 1 was 1759 with 128 children expected to have caries preventing between 258 and 348 caries at minimum and maximum effectiveness. Applying the 3 percent discount rate, the number of caries prevented during Year 1 ranged between 251 and 338. Therefore, a total of 119 and 159 additional discounted dental caries could be prevented under minimum and maximum effectiveness, respectively. Appendix III tables lists the total number of health outcomes prevented per intervention type, at minimum and maximum effectiveness for current and ideal population coverage over 10 years.

Based on our analysis, we determined that all of the interventions did reduce the number of adverse health outcomes observed in the population; however use of fluoride tooth-paste and toothbrush prevented the greatest number of caries at minimum and maximum effectiveness for the current coverage level with 1,433 and 1,910, respectively. Consequently, use of fluoride toothpaste and toothbrush also prevented the greatest number of FMDRs (159 and 211) at minimum and maximum effectiveness. Ideally, at increase coverage levels dental sealants prevented the greatest number of dental caries (3,522 and 3,870) and FMDRs (390 and 428) at minimum and maximum effectiveness.

Lastly, we determined that all interventions produced a cost savings using the cost effectiveness ratio (Equation (2.1)). While all interventions generated a cost saving, water fluoridation had the greatest cost benefit of preventing dental caries prevented ($1,335) at minimum effectiveness and dental sealants had the greatest cost benefit in preventing caries ($3,387) at maximum effectiveness over 10 years at the current coverage levels. In comparison, water fluoridation also had the greatest cost benefit in preventing caries in children receiving FMDRS ($8,149) at minimum effectiveness and maximum effectiveness ($6,053).

## Discussion

**Figure 3:**
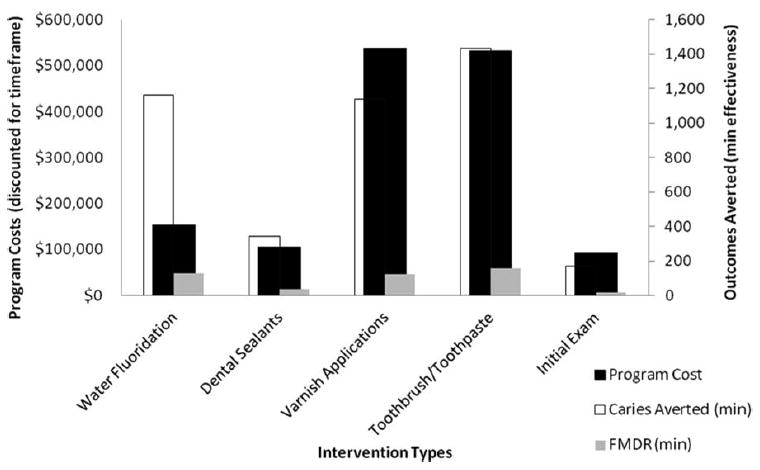
Comparison between current total program costs (discounted) and minimum number of outcomes averted (discounted) per intervention type.

In response to a request for technical assistance from the YKD dental program, we evaluated the impact of select dental interventions on the reduction of dental caries and FMDR on children aged 6-60 months. Interventions we included in our analysis were water fluoridation, dental sealants, fluoride varnish, home tooth brushing using fluoride toothpaste, and parental counseling. We chose these five interventions based on published estimates of effectiveness and research data from local YKD practicing dentist and dental providers suggesting that these interventions were most likely to have the greatest impact on the reduction of dental caries, while costing the healthcare payer the least. We found that water fluoridation, tooth brushing, and fluoride varnish would prevent the greatest number of caries and FMDRs. For instance, water fluoridation will prevent between 1,163 and 2,203 dental caries and 129-244 FMDRs. Over 10 years, the cost of supplying water fluoridation would cost $154, 610 at the current coverage level and $797,303 at ideal population coverage. However, the cost associated with preventing the caries is $1.7 million and 2.3 million at current and ideal population coverage. Thereby, saving the healthcare payer ~$1,300 for dental caries and ~7,000 for FMDRs (Appendix I). Figure 3 displays the comparison between program cost and the minimum number of health outcomes prevented. The 10-year water fluoridation program would cost considerably less than a 10-year fluoride varnish or tooth brushing program. Additionally, fluoridation of a community piped water systems would likely result in higher levels of compliance than either a dental sealant or fluoride varnish program.

One of the major limitations of this study was the lack of FMDR effectiveness data. We assumed, therefore, that the rate of effectiveness in reducing dental caries and FMDRs was essentially the same. Furthermore, we relied heavily on expert dental opinion and literature reviews to constitute whether an intervention would be effective in reducing dental caries in children. The American Academy of Pediatric Dentist and American Dental Association both agreed that using toothbrushes with fluoride toothpaste were highly beneficial in reducing the incidence of caries; however researchers believe that there is limited scientific evidence that demonstrates that fluoride toothpaste is effective in caries control in children younger than 6 years. To ensure that our results and any future evaluations are accurate and relate to specific population and its ability to reduce the number of health outcomes in the population, there is a need for more reliably available effectiveness data. For instance, dentists recommend that children should receive 8 dental sealants on their primary molars before their third birthday to protect them against dental caries and future FMDR treatment. Local dentists suggest that children’s primary molars should erupt by age 3. The published effectiveness rate for dental sealants applies only to the primary molars. Therefore, in an effort to compare sealant effectiveness to the other interventions, we had to apply a proportion for determining the number of teeth in a child’s mouth that are molars. This then provided us with the accurate effectiveness percentage for dental caries in the full mouth of a child 12-36 months of age; however this value could not be found in any literature from the YKD. Another limitation of our analysis is the use of resin-based sealants in the YKD. We used resin-based sealants to determine effectiveness for our analysis because they had a higher frequency of completion, among the YKD children, as reported by the local dental providers. However, they must to be placed on dry surfaces and young children tend to not have a dry mouth making the placement of this type of sealant extremely difficult. We understand that other types of sealants are more widely acceptable, but there use in the YKD was extremely minimal.

The evaluation for cost effectiveness was calculated using the healthcare payer perspective. We assumed that the costs associated with each dental intervention were estimated using the reimbursement fees dental providers would expect to receive from Alaska Medicaid. We assumed that all the customary administration/capital start-up and annual renewal costs were incurred through typical dentistry practice and were not covered by Medicaid; thereby costs incurred for starting and renewing interventions, with the exception of water fluoridation were not included in our analysis. We also made inferences concerning population coverage. Current population coverage was based off the annual age cohort of children 6-60 months. However, census data does not report the annual age cohort of children aged 6-12 months. Therefore, we assumed that our first age cohort was essentially half the overall birth cohort of 600 live annual births (~290). For simplicity, we assumed that all dental interventions had an ideal population coverage of 100 percent with the exception of water fluoridation; it was the only intervention in which the total number of people (1,759) that could be ideally covered was based on the number of people residing in communities capable of receiving piped fluoridated water.

Overall, we generated our analysis based solely on children residing in the YKD and thus are only repeatable in that region. Thereby, these results should not be generalized to other populations in the lower 48 states, without significant adjustments to the model. The basic model could be applied to other populations, but some of the inputs would need to be changed to make the results applicable to those specific populations. For example, the population size, the age structure, baseline caries rates, intervention effectiveness and the proportion of the population that could be served by community water fluoridation would all need to be determined.

## Conclusions

In 2008, the CDC-AIP was informed that there were high rates of dental caries requiring extensive care in the YK Delta region of Alaska. The presence of severe dental caries and the ongoing need to perform FMDRs illustrates the need of using dental interventions to reduce the prevalence of these adverse health outcomes. We evaluated which dental interventions were the most cost effective in reducing the number of carious teeth and FMDRs using the current year (2011) and over a 10-year timeframe using a simple spreadsheet-based model. All five dental interventions were shown to generate a cost saving to the healthcare payer at current and ideal population coverage using minimum and maximum effectiveness.

Overall the use of our spreadsheet-based model was useful in estimating the cost-effectiveness of these five dental interventions. However, to produce more accurate estimates for cost-effectiveness among the specific interventions, more accurate cost information, greater detail on the recommended usage for each intervention effectiveness, and greater specificity among the rate of effectiveness in reducing the number of FMDRs is required.

## Supplementary Material

3

## Figures and Tables

**Table 1:** Model Inputs for Dental Caries & FMDRS

Dental Caries	Model Inputs	Source
No. of children seen for treatment of crowns and/or fillings by a local dentist (within the dental office)	1536	YKHC Frequency of Dental Services, 2011
Annual No. children receiving 1 or more crowns	156	YKHC Quality Systems Incorporated (QSI) Electronic dental record database (“Clinical Product Suite”) , 2011 (unpublished)
Avg. No. of crowns per child*	4.54	
Annual No. of children receiving 1 or more fillings	166	
Avg. No. of teeth filled per child*	3.18	
Total number of children receiving crowns and fillings+	188	
(a)No. of children with fillings only	22	
(b)No. of children with crowns only	32	
(c)No. of children receiving crowns and/or fillings	134	
Total Avg number of crowns & fillings	7.73	
Oral Exam Cost	$66.98	FY 2012 Alaska Medicaid Reimbursement Fee Schedule.
X-Ray Cost	$89.08	
Stainless Steel Crown Cost	$199.53	
Filling Cost	$142.63	
Total Cost of Treating Crowns/Fillings (per child)#	$447	Calculated: Appendix B
**FMDRs**	**Model Inputs**	**Source**
Annual No. of FMDRs completed by dental surgeon (in operating room)	161	YKHC Frequency of Dental Services, 2011
Median No. of teeth extracted per child*	4	YKHC Quality Systems Incorporated (QSI) Electronic dental record database (“Clinical Product Suite”) ,2011 (unpublished)
Median No. Of vital pulpotomies per child*	5	
Median No. of bitewings/films taken per child*	2	
Travel cost per child (with guardian)	$1500	As reported by local YKHC dental practitioners
Personnel cost	$1500	
Operating room cost	$3198	
Stainless Steel Crown on Primary Tooth	$199.53	FY 2012 Alaska Medicaid Reimbursement Fee Schedule
Filling Cost	$142.63	
Tooth Extraction	$141.71	
Vital Pulpotomy	$131.83	
Bitewings/Films	$35	
Other Associated Costs	$250	
Total FMDR cost (per child)#	$9,349	Calculated: Appendix B

***Notes:***

* Average number of teeth being treated in the dental office was obtained from the dental clinical data maintained by the Yukon-Kuskokwim Delta Regional Health Consortium (YKHC), which uses Quality Systems Incorporated (QSI) electronic dental records named “Clinical Product Suite” to documental dental procedures and CDT billing codes to track the number and type of services rendered in the YKD.

+ Number of children receiving crowns and/or fillings in the dental office (188) was obtained through the Yukon-Kuskokwim Delta Regional Health Consortium (YKHC) Quality Systems Incorporated (QSI) electronic dental records. We then used the Venn diagram to identify and estimate the number of children that received only one type of caries service from the total number of children (i.e., 188). Thereby, the number of children receiving (a) crowns only was 32, (b) fillings only was 22, and (c) those that received a combination of crowns and fillings was 134.

# Total Costs for both caries treatment and FMDR were calculated based upon the costs associated with providing routine exams, customary procedures, and any other associated costs as detailed in the FY 2012 Alaska Medicaid Reimbursement Fee Schedule. For caries treatment in dental chair only, we calculated the mean costs using the product between the proportion of the number of children that received only one type of services (i.e., based on Venn diagram) to the total number of children receiving (188 children) and the sum of the exam and x-ray cost and the proportion between the total number of carious teeth treated per child (7.73) and AK Medicaid customary reimbursement fee for the specific type of service (i.e., cost of crown placement and fillings). All costs for FMDRs were calculated using the customary AK Medicaid reimbursement fee for each type of service.

**Table 2:** Rates of coverage and intervention effectiveness and start-up and annual running costs for 5 dental interventions.

INTERVENTION TYPES	Population Coverage		Effectiveness^a^ (%)		Unit Cost^c^($)			Source^d^
	Current Pop*	Ideal Pop*	Min	Max	Start-Up	Annual	Unit Fee	See list of references
*Water Fluoridation (6-60 months)*	11%	68%	26%	35%	$7,090	$3,625	N/A	10-13
*Dental Sealant*^b^ *(6-60 months)*	10%	100%	28%	31%	*No separate start-up and annual costs were associated with the delivery of these interventions; the costs were presumed to be included in the total program costs.*		$49.68	7,14-20
*Dental Varnish Applications (6-60 months)*	52%	100%	18%	24%			$28.50	14, 21-25
*Toothbrush & Toothpaste^[20,21]^ (6-60 months)*	55%	100%	21%	28%			$44	26-31
*\Initial Exam (6-18 months)*	26%	100%	22%	32%			$66.98	14,32,33

***Notes:***

* Current population coverage is the percentage of the population is currently receiving the intervention. Ideal population coverage is the maximum amount of coverage that could use a specific intervention. For instance, there are a total of 5 communities (36%) received community fluoridated water. However if the intervention was expanded to fluoridate all the communities that have the capacity for receiving a piped fluoridated water system (22 communities in total) the ideal population coverage will increase to 68%.

a Rate of effectiveness was obtained through expert dental opinion and literature reviews from the YK Delta Region. Sources for minimum and maximum effectiveness are provided for each intervention in the final column.

b Dental Sealants effectiveness in reducing caries in the total mouth of children upto 60 months of age was calculated using the proportion of children’s teeth that are molars (.40) and the reported effectiveness of preventing dental caries amongst the molars of children (71%-78%).

c Unit costs are composed of the start-up and annual costs, along with the unit fee associated with each intervention. Water fluoridation was the only intervention by which start-up and annual costs were a part of the total intervention/program costs. The costs associated with water fluoridation consist of $5,970 in start-costs ($4,380 for installation and $1,590 for an operator’s travel to the community) and $2,384 in annual costs ($1,120 for the operator’s fee above their own salary and $1,264 for annual renewal). In all other interventions, we assumed that the start-up and annual costs were paid for as part of their program cost. Thus, their costs were presumed to be included as part of the regular administration/capital costs of the YK region. Thus, the only cost associated with these other interventions is the unit cost, per tooth or per visit, associated with performing the service. The unit fee was obtained from the License for use of CDT Codes, FY 2012 Dental Fee Reimbursement Schedule [5].

d These numbers represent the sources for effectiveness data. A full list of sources is available in the reference section.

**Table 3:** A: Estimated annual costs of each dental intervention

Intervention Type	Annual undiscounted program costs*		Annual discounted program cost (one year only)+		Total discounted program costs (10 years)+	
	Current Pop**	Ideal Pop**	*Current Pop*	*Ideal Pop*	*Current Pop*	*Ideal Pop*
Water Fluoridation	$18,125	$200,280	$17,597	$194,447	$154,610	$797,303
Dental Sealants	$12,420	$226,938	$12,058	$220,328	$105,945	$1,935,829
Fluoride Varnish	$62,923	$146,775	$61,090	$142,500	$536,747	$1,252,021
Toothbrush/Toothpaste	$62,315	$113,000	$60,500	$110,000	$531,560	$966,472
Initial Exams	$10,851	$38,179	$10,535	$37,067	$92,559	$325,671

B: Comparison of Annual (undiscounted Year 1) estimates for each intervention at minimum effectiveness					
Outcome Measure	**Water Fluoridation**	**Dental Sealants**	**Fluoride Varnish**	**Toothbrush/Toothpaste**	**Initial Exams**
**# Children Covered**	929	250	1311	1416	162
**# children w/caries**^a^	65	18	95	103	11
**Total # of caries**^a^	525	141	740	799	91
**% Effectiveness**	.26	.71	.18	.21	.22
**# of Caries Averted**^b^	137	39	133	168	20
**Annual Program $**^c^	$18,125	$12,420	$62,923	$62,315	$10,851
**Annual Averted $**^d^	$200,245	$58,833	$195,347	$246,158	$29,503
**Program $ per child**^c^	$278	$690	$662	$605	$986
**Averted $ per child**^d^	$3,081	$3,269	$2,056	$2,390	$2,682

***Notes:***

* Annual estimated costs are the calculated undiscounted costs determined from the actual cost of supplying the intervention and any additional costs associated with the implementation of the intervention into the population.

** Current pop (population) is defined as the current (‘present day”) number of people receiving the intervention. Ideal population is defined as the maximum number of people that could benefit from the use of the intervention. The cost for each population type is calculated using the product between population coverage rates and the intervention unit costs (Table 2).

+ Discounted costs are the actual present value (PV) of implementing the specific intervention to the population for the selected timeframe based upon the intervention specific estimated (i.e. budgeted) costs. All discounted costs are calculated as Current $/(1+discount rate)ˆspecified timeframe.

a # of children with caries is calculated by multiplying the population by caries incidence (7.3%), whereas the # of caries is calculated by multiplying the number of children with caries by the mean number of carious teeth per child (7.73).

b # of caries averted is the product between the total number of caries and the percentage of effectiveness. Hint: the % effectiveness for dental sealants is multiplied by .40 to to obtain the actual % effectiveness of preventing carious teeth amongst the total mouth of children (~20 teeth). The effectiveness value shown only represents that of permanent molar teeth and not the whole mouth.

c Annual program cost was calculated earlier (See Methods: Section D for Equation 2.4). This is the undiscounted cost for using the intervention to treat children with carious teeth. The program cost per child is calculated by dividing the annual program cost by the number of children with caries.

d Annual averted cost is the product between the number of caries averted and the cost of treating caries ($1,467 per child). The averted cost per child is the annual averted cost divided by the number of children with caries.

**Table 4:** Comparison of annual (undiscounted Year 1) estimates for each intervention at minimum effectiveness

Outcome measure	Water fluoridation	Dental sealants	Fluoride varnish	Toothbrush/toothpaste	Initial exams
# Children covered	929	250	1311	1416	162
# Children w/caries*	65	18	95	103	11
Total # of caries*	525	141	740	799	91
% Effectiveness	0.26	0.71	0.18	0.21	0.22
# of caries averted†	137	39	133	168	20
Annual program $‡	$18,125	$12,420	$62,923	$62,315	$10,851
Annual averted $§	$200,245	$58,833	$195,347	$246,158	$29,503
Program $per child‡	$278	$690	$662	$605	$986
Averted $per child§	$3,081	$3,269	$2,056	$2,390	$2,682

* # of children with caries is calculated by multiplying the population by caries incidence (7.3%), whereas the # of caries is calculated by multiplying the number of children with caries by the mean number of carious teeth per child (7.73).

† # of caries averted is the product between the total number of caries and the percentage of effectiveness. Hint: the % effectiveness for dental sealants is multiplied by .40 to obtain the actual % effectiveness of preventing carious teeth amongst the total mouth of children (320 teeth). The effectiveness value shown only represents that of permanent molar teeth and not the whole mouth.

‡ Annual program cost was calculated earlier (See Methods: Dental Intervention Program Cost section for Equation (2.1)). This is the undiscounted cost for using the intervention to treat children with carious teeth. The program cost per child is calculated by dividing the annual program cost by the number of children with caries.

§ Annual averted cost is the product between the number of caries averted and the cost of treating caries ($1,467 per child). The averted cost per child is the annual averted cost divided by the number of children with caries.

## Appendix I: Actual tool

Spreadsheet-based model used to evaluate the economic impact of six dental interventions used to reduce the number of dental caries and FMDRs among Alaskan Native children in the YK Delta Region.

## Appendix II: Mean cost calculations

1. **Crown $** = (# of children receiving crowns only/Total # children receiving crown, filling, or both) × ((Oral Exam $ + X-Ray $) + (Avg # of crowns & fillings per child × Crown Reimbursement $))
  ***Where:*** # of children w/crowns only = 22
    Total # of children receiving crowns, fillings, or both = 188
    Oral Exam $=$66.98
    X-Ray $= $89.08Avg # of crowns & fillings = 7.73
    Crown Medicaid Reimbursement $= $199.53
2. **Filling $** = (# of children receiving fillings only/Total # children receiving crowns, fillings, or both) × ((Oral Exam $ + X-Ray $) + (Avg # of crowns & fillings per child × Filling Reimbursement $))
  ***Where:*** # of children w/crowns only = 32
    Total # of children receiving crowns, fillings, or both = 188
    Oral Exam $=$66.98
    X-Ray $= $89.08
    Avg # of crowns & fillings = 7.73
    Filling Medicaid Reimbursement $= $142.63
3. **Crown & Filling $** = (# of children receiving crown & Fillings/Total # children receiving crown, filling, or both) × ((Oral Exam $ + X-Ray $) + (Avg # of crowns & fillings per child × percent of crowns to fillings × Crown Reimbursement $) + (Avg # of crowns & fillings per child × percent of fillings to crowns × Filling Reimbursement $))
  ***Where:*** # of children w/crowns only = 134
    Total # of children receiving crowns, fillings, or both = 188
    Oral Exam $ =$66.98
    X-Ray $ = $89.08
    Avg # of crowns & fillings = 7.73
    Percent of crowns & fillings = 50 percent
    Crown Medicaid Reimbursement = $199.53
    Filling Medicaid Reimbursement $ = $142.63
4. **Treatment Cost per Child** = Crown Mean Treatment $ + Filling Mean Treatment Cost + Crown & Filling Treatment Cost
  ***Where:*** Mean Crown Cost is $199 (Equation 1 above)
    Mean Filling Cost is $214 ((Equation (2.1) above)
    Mean Cost for both is $1,050) (Equation 3 above)
5. **FMDR Treatment Cost per child** = Sum of all procedural costs associated with performing caries treatment under general anesthesia in an operating room. All costs are provided in Table 1.

**Table:** Weighted cost values

Weighted data inputs (per child)	Mean cost calculations
Crowns only	$199
Fillings only	$214
Crowns & fillings	$1,050.17
Mean treatment cost with caries	$1,461.73
Mean treatment cost with FMDR	$9,349

## Appendix III

**Table:** Discounted number of dental caries & FMDR procedures prevented at minimum effectiveness at current & ideal population coverage (per intervention type)

Intervention type	Caries prevented (Year 1, Year 10)				FMDR prevented (Year 1, Year 10)			
	Current Caries		Ideal Caries		Current Procedures		Ideal Procedures	
Water Fluoridation	132	**1,163**	251	**2,203**	15	**129**	28	**244**
Dental Sealants	39	**342**	401	**3,522**	4	**38**	27	**235**
Fluoride Varnish	129	**1,137**	254	**2,233**	14	**126**	28	**247**
Toothbrush/ toothpaste	163	**1,433**	296	**2,605**	18	**159**	33	**288**
Initial Exam	20	**172**	242	**2,125**	2	**19**	27	**235**

**Table:** Discounted number of dental caries & procedures requiring general anesthesia prevented at maximum effectiveness at current & ideal population coverage

Intervention type	Caries prevented (Year 1, Year 10)				FMDR prevented (Year 1, Year 10)			
	Current Caries		Ideal Carie		Current Procedures		Ideal Procedures	
Water Fluoridation	178	**1,566**	338	**2,965**	20	**173**	37	**328**
Dental Sealants	43	**376**	440	**3,870**	5	**42**	49	**428**
Fluoride Varnish	1,72	**1,516**	339	**2,977**	19	**168**	38	**330**
Toothbrush/ toothpaste	217	**1,910**	395	**3,473**	24	**211**	44	**385**
Initial Exam	28	**250**	352	**3,090**	3	**28**	39	**342**
